# Editorial: Bioaccessibility of food-derived bioactive components and their health-promoting mechanisms

**DOI:** 10.3389/fnut.2023.1191671

**Published:** 2023-05-17

**Authors:** Tian Ren

**Affiliations:** College of Food Engineering and Nutritional Science, Shaanxi Normal University, Xi'an, China

**Keywords:** bioactive ingredient, health-promoting, isolation, identification, toxicology, modes of action

With the growth of research related to bioactive components in food and their biological activities, more detailed studies with innovative methods and convincing data are needed to further clarify bioactivities, evaluate their safety risks, and reveal the mechanisms prior to the widespread application of these components in the food industry. Therefore, this Research Topic has been proposed to gather original research or reviews on the effects of food-derived bioactive ingredients and their health-promoting mechanisms for the purpose of their extensive use for food production and human health.

This Research Topic brought together important research contributions on the isolation and identification of bioactive components in food. Ma et al. identified 18 phenolic compounds dominated by the derivatives of quercetin and myricetin from miracle berry (*Synsepalum dulcificum*) leaf extract by UPLC-DAD-MS, contributing its anti-angiogenesis and anticancer activities. Chen H. et al. screened new peptide with high angiotensin-I-converting enzyme inhibitory activity from oyster (Crassostrea gigas) hydrolysates obtained from simulated gastro-intestinal digestion and followed by isolation through RP-HPLC. Wu et al. investigated chemical compositions, particularly for phytochemical contents with anti-aging effects of standardized herbal chicken essence through HPLC. Chen Y.-N. et al. extracted antimicrobial peptides from sturgeon (*Acipenser schrencki*) spermary proteins. The molecular weight and hydrolytic amino acid composition and content of the peptides were characterized by Tris-SDS-PAGE and amino acid analyzer, respectively.

Plenty of research has evaluated the health benefits of bioactive ingredients by using cells, mice, or rats models. In this gathering, Wei et al. demonstrated that two triterpenoids, Hxrarg and Hxra, extracted from *Nigella sativa* seeds improved blood glucose control by enhanced beta cells activity and alleviation of insulin resistance. The related mechanisms were systematically explained by an IR-3T3-L1 cell model and Western blot analysis. Chen G. et al. investigated the effects of chlorantraniliprole on lipogenesis in 3T3-L1 cells by transcriptomics of RNA-seq and concluded that chlorantraniliprole may affect the pathways related to adipocyte differentiation and circadian rhythm to promote adipogenesis. Ma et al. accessed the anticancer activity of miracle berry leaf extract by using a xenograft mouse model. Chen H. et al. evaluated the bioactivity of the novel peptide through hypertensive rats, and the mechanisms of peptide on the high angiotensin-I-converting enzyme inhibitory activity were illustrated by the molecular docking method. Wu et al. investigated the anti-aging effects of standardized herbal chicken essence on D-galactose-induced aging mice, with further confirmation through Western blot analysis. Xu et al. investigated the use of the traditional Chinese medicine Tongxie Yaofang to treat irritable bowel syndrome with diarrhea and type 2 diabetes mellitus in rats with live depression and spleen deficiency. In addition to traditional mice models, Ma et al. further assessed the toxicity and ant angiogenesis activity of miracle berry leaf extract by using a zebrafish embryo model.

This Research Topic compiles seven peer-reviewed articles conveying several relevant points regarding the isolation and identification of bio-based extracts and compounds, toxicology, the assessment of health effects, and mechanisms of action. Some of the chemicals, such as the Chinese medicine Tongxie Yaofang, need to be further studied to investigate their active compounds. Overall, we hope these articles provide basic information for related industries, offer new insights for the progress of the food industry, and inspire scientists to push advances in this area even further. We would like to express our great gratitude to the contributors for providing up-to-date and high-quality original articles. We are also very grateful to the editorial team and reviewers for their support and excellent cooperation.

## Author contributions

TR contributed to wrote the first draft of the manuscript and approved the submitted version.

